# Parenchyma-Sparing Liver Resection or Regenerative Liver Surgery: Which Way to Go?

**DOI:** 10.3390/medicina58101422

**Published:** 2022-10-10

**Authors:** Florin Botea, Alexandru Bârcu, Alin Kraft, Irinel Popescu, Michael Linecker

**Affiliations:** 1 Faculty of Medicine, “Titu Maiorescu” University, 031593 Bucharest, Romania; 2 “Dan Setlacec” Center of General Surgery and Liver Transplantation, Fundeni Clinical Institute, 022328 Bucharest, Romania; 3 Department of Surgery and Transplantation, UKSH Campus Kiel, 24105 Kiel, Germany

**Keywords:** liver resection, therapeutic options in liver surgery, regenerative liver surgery, parenchyma-sparing liver resection

## Abstract

Liver resection for malignant tumors should respect oncological margins while ensuring safety and improving the quality of life, therefore tumor staging, underlying liver disease and performance status should all be attentively assessed in the decision process. The concept of parenchyma-sparing liver surgery is nowadays used as an alternative to major hepatectomies to address deeply located lesions with intricate topography by means of complex multiplanar parenchyma-sparing liver resections, preferably under the guidance of intraoperative ultrasound. Regenerative liver surgery evolved as a liver growth induction method to increase resectability by stimulating the hypertrophy of the parenchyma intended to remain after resection (referred to as future liver remnant), achievable by portal vein embolization and liver venous deprivation as interventional approaches, and portal vein ligation and associating liver partition and portal vein ligation for staged hepatectomy as surgical techniques. Interestingly, although both strategies have the same conceptual origin, they eventually became caught in the never-ending parenchyma-sparing liver surgery vs. regenerative liver surgery debate. However, these strategies are both valid and must both be mastered and used to increase resectability. In our opinion, we consider parenchyma-sparing liver surgery along with techniques of complex liver resection and intraoperative ultrasound guidance the preferred strategy to treat liver tumors. In addition, liver volume-manipulating regenerative surgery should be employed when resectability needs to be extended beyond the possibilities of parenchyma-sparing liver surgery.

## 1. Introduction

Liver resection (LR) for malignant tumors should respect oncological margins while ensuring safety and improving the quality of life [1]. Tumor staging, underlying liver disease and performance status should all be attentively assessed in the decision process for LR [1].

Tumors should be regarded as resectable when negative margins and an adequate future liver remnant with preserved in- and outflow, as well as biliary drainage, can be achieved, irrespective of the anatomic location within the liver or relation to the key vascular and biliary structures [2]. The form of surgery that preserves as much liver parenchyma as possible is known as parenchyma-sparing surgery (PSS). It is probably the most popular, and nowadays the first-choice strategy for removing liver tumors in general. The use of enhanced techniques and intraoperative ultrasound guidance significantly increases precision of resection and thereby resectability [3]. On the other hand, regenerative surgery (RS) has evolved as a competitive strategy to increase resectability by augmenting the future liver remnant, often excluding PSS at the same time. This situation has led to a long-lasting debate between advocates of one or the other strategy. The present paper reviews both strategies, analyzing their current role in liver surgery with a special focus on their synergistic qualities.

## 2. Parenchyma-Sparing Liver Surgery

PSS was first used in small superficial colorectal liver metastases (CRLMs), as an indication for minor anatomic LR (“cherry-picking surgery”) [4,5,6]. Deeply located lesions with intricate topography, which at first were an indication for major anatomic LR, can now be addressed by complex multiplanar parenchyma-sparing (PS) LR, preferably under the guidance of intraoperative ultrasound (IOUS) [7,8,9,10].

If achievable, single or multiple PS LRs are preferred over upfront major LR with wider oncological margins, regardless of the tumor extension [1], as there is no difference in the oncological results [11]. PS LR was proved to have the same oncological benefit as major hepatectomies and was associated at the same time with a better safety profile [12,13,14].

PSS can be used as alternative to major LR for removing lesions from major vessels, even allowing LR with 0 mm vascular margins (R1vasc) in deeply located tumors, otherwise surgically unmanageable [1]. Compared to staged LR, R1vasc has better results in terms of safety [3,15], eliminates the drop-out risk, has comparable recurrence and permits greater salvageability [16,17]. Partial resection and vein reconstruction are options for HV invasion [18].

IOUS aids PSS as it facilitates locating the tumor and assessing its relationship with biliary and vascular structures. Moreover, IOUS accurately guides the transection plane to obtain either R0 surgical or R1vasc LR [19,20]. IOUS Doppler flow analysis detects distal collateral veins (CVs) between HVs and evaluates inflow trajectory after HVs have been clamped [8,9,10]. As distal CVs ensure a sufficient outflow, liver parenchyma can be spared even if the main hepatic vein (HV) is sectioned [8,9,10]. CVs can be preoperatively assessed by imaging techniques, yet IOUS color-flow analysis can better determine their patency [1]. HV clamping helps by increasing CV patency, with persistent hepatopetal inflow allowing for PSS despite CVs being unapparent [7,8].

PSS now comprises various techniques: single or multiple wedge LR, anatomic or non-anatomic hepatectomies of one or two liver segments or subsegments, anatomic bisegmentectomies and complex LR for deeply located tumors in contact with major vessels, such as [21,22,23]:

- systematic extended right posterior sectionectomy, as an alternative to right hemi-hepatectomy [24]—segment (S) 6–7 resection partially extended to S5 and/or S8 with right HV division; middle HV branches supply outflow of preserved S5 and/or S8;
- mini-upper transversal hepatectomy, as an alternative to right hemi-hepatectomy—S7–8 anatomic or limited resection with right HV division; inferior right HV [25], middle HV branches or distal CVs between right and middle HVs supply outflow of S5–6 [10];
- right upper transversal hepatectomy, as an alternative to right extended hemi-hepatectomy [26]—S7–S8–S4 superior anatomic or limited resection with right and middle HV division; inferior right HV and/or distal CVs between right, middle and left HVs supply outflow of S4 inferior–5–6;
- left upper transversal hepatectomy, as an alternative to left extended hemi-hepatectomy [10]—S2–S4 superior or S2–S4 superior–S8 anatomic or limited resection with left HV or left and middle HV division; distal CVs between left, middle and/or right HV supply outflow of S3–4 inferior–5;
- total upper transversal hepatectomy [10]—S2–S4 superior–S7–S8 anatomic or limited resection with right, middle and left HV division given the existence of an inferior right HV and CVs between hepatic HVs stumps, that provide outflow of S3–S4 inferior–S5–S6;
- mini-mesohepatectomy, as an alternative to central hepatectomy [27]—S4 superior–S8 anatomic or limited resection with middle HV division; distal CVs between middle HV and right and left HVs supply outflow of S5–S4 inferior;
- liver tunnel, as an alternative to central hepatectomy plus S1 segmentectomy [10,28]—S8 anatomic or limited resection with complete S1 removal;
- liver tunnel extended to segment 4 superior, as an alternative to central hepatectomy plus S1 segmentectomy [10,28]—S4 superior–S8 anatomic or limited resection with complete S1 removal and middle HV division; distal CVs between middle HV and right and left HVs supply outflow for S5–S4 inferior;
- systematic limited central, as an alternative to central hepatectomy—sparing the portal pedicle (P) for S8 dorsal and some of P4 and/or P5 pedicles (depending on tumor location). IOUS guides the right transection plane along the P8 dorsal, intersecting the P8 ventral and as few P5 pedicles as possible. The left transection plane is settled relative to tumor position between Cantlie’s line and the falciform ligament [29];
- left anterior sectorectomy, as an alternative to left hepatectomy for lesions invading the distal part of the umbilical portion of the left portal vein–resection of S3 and S4 inferior, while preserving the P2 and P4 superior.

## 3. Regenerative Liver Surgery

Regenerative surgery (RS) evolved as a method to increase resectability by stimulating the hypertrophy of the parenchyma intended to remain after resection, which is referred to as future liver remnant (FLR) [30]. This liver growth induction can be achieved by portal vein embolization (PVE) and liver venous deprivation (LVD) as interventional approaches, and portal vein ligation (PVL) and associating liver partition and portal vein ligation for staged hepatectomy (ALPPS) as surgical techniques.

PVE was conceived by Makuuchi et al. in 1990 as a tool to induce hypertrophy of the FLR and decrease the risk of liver failure after major hepatectomy, enabling major anatomical LR, which would otherwise not be feasible [31]. The mechanism behind this approach is based on the redirection of the portal flow, that stimulates the contralateral hypertrophy. PVE is associated with a low morbidity and mortality. However, the growth of FLR is limited to a volume by 40% at best, for most cases within a period of around 2 months [32]. This may lead to insufficient FLR and/or tumor progression while waiting for hypertrophy. PVL (open or minimally invasive surgery) is a feasible alternative to PVE. For patients undergoing PVE, major hepatectomy becomes feasible in 2/3 of cases with a similar overall survival to those without PVE [33]. Chemotherapy after PVE decreases the tumor progression rate and has not been shown to decrease liver hypertrophy. In about 1/3 of patients, PVE fails and leads to canceling of the planned LR (drop-out rate) [34].

Recently, liver venous deprivation (LVD), consisting of embolization of both the PV and one or two HVs of the hemi-liver, has been proposed as a promising way for improved regeneration (1–2 weeks) [35,36]. Several studies comparing LVD to PVE reported improved FLR volume growth following LVD [37,38,39], as well as better FLR functional regeneration [40]. In particular, one study has shown a more than 75% increase in the kinetic growth rate of the FLR after LVD compared to PVE [35]. Moreover, a 54% functional increase in the FLR 7 days after LVD has been reported [40]. However, literature data on LVD of a cirrhotic liver are lacking [41]. 

The two-stage hepatectomy (TSH) was introduced in 2000 as two successive surgical steps for removing multiple bilobar tumors that cannot be removed by a sole hepatectomy [42]. Usually, the response to neoadjuvant chemotherapy was used to select candidates with favorable tumor biology. TSH can be used by itself or combined with PVE or portal vein ligation (PVL) [43]. It usually has resection rates of up to 70–75%; the main reason for non-completion is disease progression between the two stages (around 90% of cases) [42,44]. The postoperative morbidity rate is around 20% after the 1st stage and 40% after the 2nd stage, with an overall mortality below 5% [45].

In 2012, Schnitzbauer et al. proposed combining PVL with in situ liver partition to obtain rapid FLR hypertrophy (in 7–10 days) as a new strategy to increase resectability [46], which was subsequently termed ALPPS [47]. One mechanism behind this technique is thought to trigger an inflammatory response that induces a growth rate of 22–35 mL daily, significantly superior to PVE (3–5 mL daily) [48]. However, this volume growth does not automatically equal an increase in liver function [49]. This strategy results in a FLR increase of up to 80% and above (compared to 40% in PVE/PVL), while shortening the interstage period to 1–2 weeks [50]. Moreover, ALPPS enables resection rates to increase to more than 90% [51,52,53], now being feasible even when using a minimally invasive approach [54].

However, especially during early phase of this technique, the postoperative mortality rate was up to 15% [46,55]. The first reported morbidity rate was 64%, out of which 44% events were Clavien–Dindo grade III or IV [46]. To improve the results, a series of modifications were proposed, as follows:

- Delayed ALPPS. The interstage interval from the first to the second step surgery was extended from 7–9 days to 14–21 days to give the FLR time for functional recovery, which resulted in an important decrease in the postoperative morbidity [56,57].
- Partial ALPPS. Partial parenchymal transection (50% to 80%) during the first step of surgery [58] avoids complications linked with complete transection (such as bleeding, bile leak and infectious complications of ischemic segment 4). It reduced both morbidity and mortality compared to the conventional technique, while still resulting in FLR hypertrophy of at least 50% [58,59].
- Segment 4 portal pedicle-sparing ALPPS. Preserving the main portal pedicles of S4 during parenchyma transection in the first step avoids local ischemia [60].
- PVE-ALPPS. Allows avoiding dissection of the hilum in the first step of surgery, needed for the ligation of the right portal vein. “No touch” techniques using PVE have been proposed.
- Transhepatic right portal vein (RPV) approach, pre- or intraoperatively (hybrid ALPPS) [61].
- RPV approach via the inferior mesenteric (mini-ALPPS) [62] or ileocecal portal vein (TIPE ALPPS/ALPTIPS) [63].

Of note, although relatively easy to perform, tourniquet ALPPS [64] might be associated with a higher risk of operative events during the second stage due to severe adhesions/perihilar fibrosis.

Short-term results after ALPPS, that were initially a major concern, have been continuously improved over time, now reaching 90-day mortality rates below 5% [65] and a relatively low major morbidity (21%) in high-volume centers.

**Figure 1:**
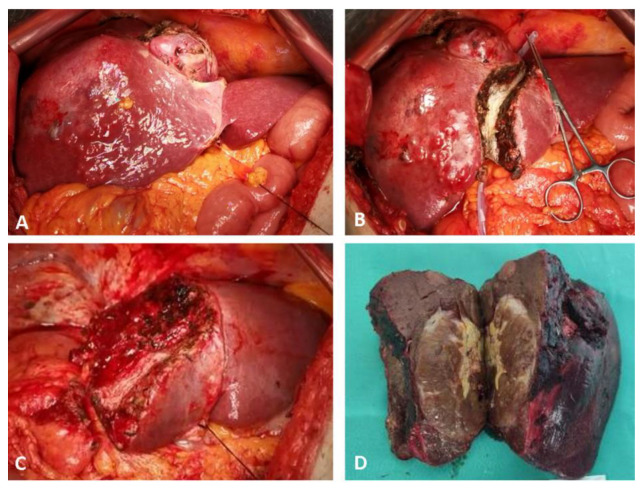
Intraoperative aspects of parenchymal sparing ALPPS in a 67-year-old male patient, for a large HCC located in segments 4, 5, 6, 7 and 8, with satellites in segment 4, on HBV chronic hepatitis. Stage 1: (**A**) intraoperative aspect at exploration; (**B**) ultrasound-guided partitioning of the liver through segment 4, adding the non-tumoral parenchyma of segment 4 to the FLR. Stage 2 after an interstage interval of 14 days; (**C**) remnant liver after completion of right hemi-hepatectomy non-anatomically extended to segment 4; (**D**) surgical specimen. No intraoperative adverse events were encountered during both operations, and only minor ascites after stage 2 were recorded as complications.

**Figure 2:**
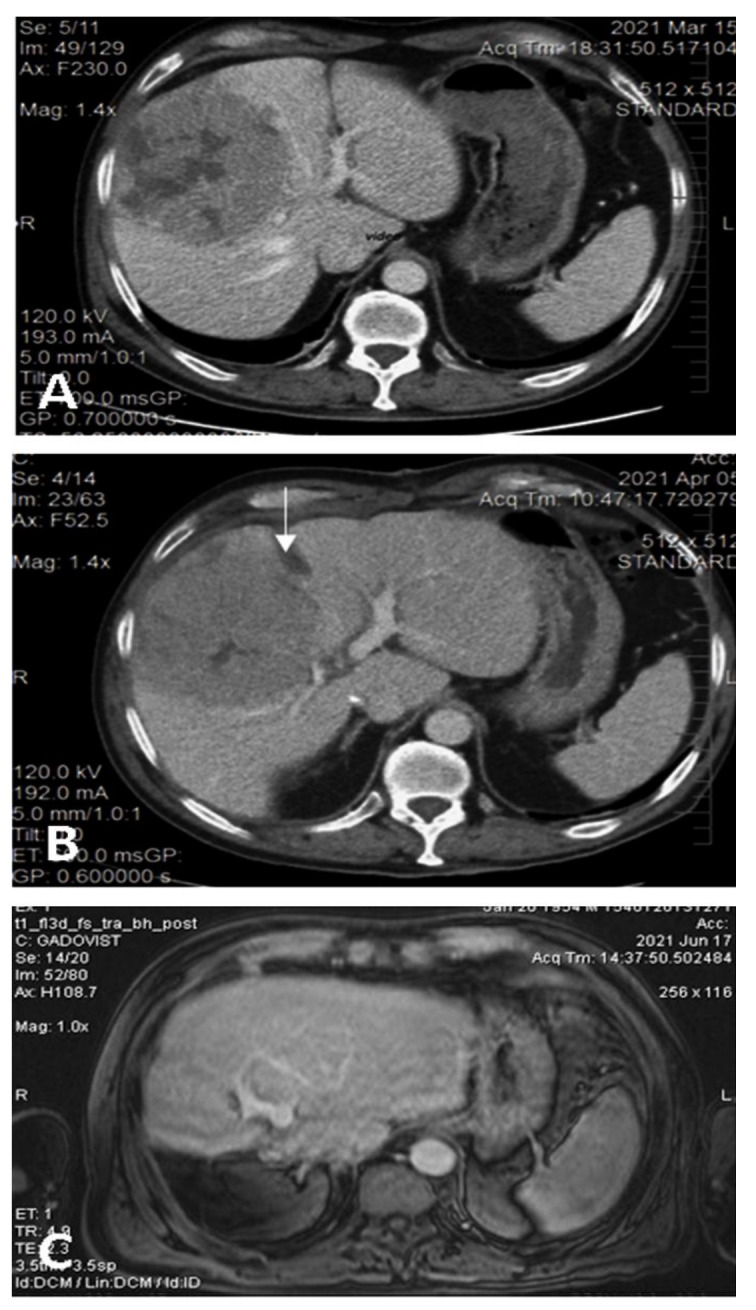
(**A**). Preoperative CT showing the large HCC located in segments 5 and 8 with extension to segments 6, 7, 4, compressing the middle hepatic vein; volumetry: volume of segments 2 and 3 16.8% of total functional liver volume, volume of FLR 27.8%. (**B**) Interstage CT showing the liver partitioning, absence of contrast in the right portal vein (due to ligation), and sufficient growth of FLR (38.5% of total functional liver volume). (**C**) Postoperative CT with well-perfused, non-dilated bile ducts, and tumor-free remnant liver.

To further increase resectability while reducing morbidity, we proposed a new technical variant of ALPPS—parenchyma-sparing ALPPS (psALPPS)—that involves shifting the transection plane through segment 4 using IOUS guidance, preserving part of this segment along with the left lateral section [66]. Besides avoiding S4 necrosis, that is a source of complication when performing conventional ALPPS, a significant advantage of psALPPS lies in preventing major bile leaks at the transection surface by avoiding complete exclusion of S4 from the biliary system (as in conventional ALPPS). Parenchyma-sparing ALPPS offers the advantage of maximizing FLR while simultaneously reducing ischemic injury of S4 compared to conventional ALPPS (Figure 1 and Figure 2). Moreover, when compared to standard ALPPS, partitioning through segment 4, away from the umbilical portion of the left portal pedicle, protects against potential injuries of the vascular and biliary structures for segments 2 and 3. This new technical variant also embeds some of the main modifications already proposed, such as partial ALPPS, avoiding the transection beyond the middle HV, and delayed ALPSS [66]. It also adapts the concept of avoiding hilar dissection by adopting a minimal hilar dissection (right side approach only) [66]. Using an extra-Glissonean approach to complete the hepatectomy during the second step further increases safety by avoiding re-dissection of the liver hilum.

Besides being the best available tool for identifying and mapping the focal liver lesions in real time, IOUS increases the safety of ALPPS and its variants by identifying the anatomic variants of the portal vein bifurcation and guiding the right portal vein ligation and the liver transection.

## 4. Parenchyma-Sparing vs. Regenerative Liver Surgery

Interestingly, both strategies have the same origin, as they were basically devised by Makuuchi et al., who pioneered PSS [25] by promoting ultrasound-guided LR and RS by devising portal vein embolization [31]. Even though “siblings”, they become “enemies” caught in the PPS vs. RS debate. However, these strategies are both valid and must be both mastered and used to increase resectability.

A major advantage of PSS lies in the lower rate of complications, including postoperative acute liver failure, due to an insufficient FLR [12], thereby making PSS a useful tool for preventing the “small-for-size” syndrome, while still safe from an oncological standpoint [13,14].

Therefore, PSS should be the first-choice strategy to apply to ensure resectability, with RS as an alternative whenever PSS is considered not feasible. However, the PSS feasibility varies a lot with the expertise and willingness to deploy specific surgical techniques and use of intraoperative ultrasound guidance. Therefore, the more the PSS is implemented, the fewer the cases for RS, and vice versa. In case of extensive tumors, PSS can be successfully used to ensure resectability, avoiding RS that results in tumor progression [67] and more postoperative complications [46]. Given the superiority of PSS in terms of safety, this strategy should be favored over RS. Ideally, PSS with all its enhancements should be the main approach for LR, leaving RS to ensure resectability only in patients where PSS is not feasible. Obviously, independently of surgical strategy, neoadjuvant chemotherapy is mandatory to control advanced liver tumors.

Therefore, the two strategies should not compete, but rather complement each other in a coherent treatment protocol.

PsALPPS combines both concepts of complex liver surgery, RS and PSS, which synergistically achieve resectability, which would otherwise not be possible with either approach [66].

The minimally invasive approach is feasible for both PSS and RS. However, for complex bilobar deeply located liver tumors, PSS is often not feasible due to technical limitations of this approach, making the RS approach, even ALPPS, a technical alternative. Nevertheless, the type of approach should not change the indication for a certain resection strategy. Therefore, if complex PSS is indicated, this should be carried out even if it is feasible only by open approach, and not switched to major two-stage LR only to perform laparoscopic surgery.

### 4.1. Colorectal Liver Metastases

Downstaging of initially unresectable CRLM may be achieved using novel cytotoxic and biologic systemic therapy to achieve curative surgery with best results when carried out in tertiary referral centers with an expert multidisciplinary team [68,69].

Compared with non-PSS, perioperative outcomes are better in the case of PSS, with PSS also being associated with satisfactory oncological results. By sparing liver parenchyma, PSS allows repeat hepatectomy in the likely event of liver recurrence. PSS also showed beneficial OS and RFS rates [11]. Matsumura et al. showed that, in advanced CRLM, PS LR was not associated with more positive surgical margins or local recurrence when compared with major LR [70]. Mise et al. showed that PS LR impacts OS, RFS or liver-only RFS by allowing repeat LR, with similar perioperative morbimortality [71], while not increasing the local recurrence risk [17]. Neither survival, recurrence risk or site are influenced by the extent of a negative surgical margin [72]. In the setting of modern chemotherapy, Adam et al. demonstrated that R1 margins may yet be linked with similar OS [73]. The R1vasc approach is safe concerning oncological results in CRLM [74], yet hepatectomy en bloc with a vascular element is the preferable approach, given the confirmed true vascular invasion [18].

When compared to major LR, PS LR was linked to lower overall morbidity, fewer major complications and a shorter hospital stay while no significant differences were observed for postoperative liver insufficiency and positive resection margins [75]. The tumor burden (with a score ≥4.5) is related to a higher rate of positive resection margins both in major and PS LR [75]. Independently of tumor burden, the 5-year OS and RFS were similar for PS LR [75].

RS, such as PVE [76,77] and TSH [46,78], has made it possible for more patients with colorectal liver metastases (CRLMs) to benefit from curative LR [79,80]. Unresectable CRLMs are often related to a difficulty in completely removing all lesions and preserving as FLR at least two contiguous functional segments [81]. CRLM is the most common indication for ALPPS, with reported OS rates of 28–54% at 3 years, and of 32–58% at 5 years [82].

When comparing ALPPS with TSH for CRLM, a superior increase in volume was proved, with a shorter median necessary time to obtain it for ALPPS (8 vs. 28 days). However, the overall morbidity was higher for ALPPS (58.3% vs. 11.1% for stage 1 and 83.3% vs. 38.2% for stage 2). The 1-year OS rates were similar and the DFS rate was higher in TSH (80% vs. 67%) [83]. Nevertheless, if performed at high-volume centers in selected patients, data suggest its superiority to TSH [51,84]. In a study on 100 patients with CRLM and sFLR <30%, patients randomized to undergo ALPPS had higher resection rates (92% vs. 80%) and better OS (46 vs. 26 months) than those having TSH, with no significant differences in morbidity, 90-day mortality or R0 resection rates [51,84].

A systematic review of a TSH series [45] showed that despite satisfactory OS and DFS rates with acceptable morbidity and mortality, a significant number of patients (8–31%) did not finish the procedure. The main dropout reasons were disease progression during the interval (88% of the dropout) and insufficient FLR hypertrophy (4% of the dropout) [45,85].

Both ALPPS and TSH are effective strategies for achieving R0 LR with favorable impact on long-term prognosis [80] even for patients with underlying liver impairment after prolonged chemotherapy that is often required to convert patients to a resectable status [80,86]. The advantage of ALPPS in terms of feasibility compared with TSH is real but limited to when the procedure is clearly indicated [83].

A study on laparoscopic PS LR for CRLM has shown a low postoperative morbidity rate (14.5%), low conversion rate (1.7%) and no postoperative mortality; for the majority of patients (81%), R0 LR was obtained and the 5-year OS and RFS were similar to open surgery rates [87].

When comparing laparoscopic bilobar and single LR, the conversion rate, R0 resection rate and need for transfusion are similar. Bilobar LRs, out of which most were major hepatectomies, were associated with longer operative time and hospital stay as well as higher blood loss. However, both single and bilobar LRs were linked to no postoperative mortality, comparable major morbidity (<5%), recurrence risk, OS and RFS [88].

### 4.2. Hepatocellular Carcinoma

There are several curative options for the treatment of hepatocellular carcinoma (HCC), e.g., usually intraoperative or percutaneous tumor ablation (for tumors <3 cm), hepatectomy and liver transplantation, particularly in the setting of advanced cirrhosis [89]. Targets of LR are improving recovery, reducing postoperative morbidity and ensuring a satisfactory function of a frequently cirrhotic liver [89]. R0 resection is no longer considered an absolute requirement, as R1 resection has become an accepted option for encapsulated HCC in contact with major vascular and biliary structures [90]. Hasegawa et al. proved that anatomical LR is oncologically superior to non-anatomical LR [91]. As anatomical LR included segmentectomies and sub-segmentectomies [91], we underlined that this strategy is also a parenchyma-sparing one. However, other studies showed no differences regarding oncological outcomes between anatomical and non-anatomical LR [92,93,94].

PS LRs are especially beneficial in case of cirrhosis. When compared to the right posterior sectionectomy, the right hepatectomy for HCC was more frequently associated with liver failure (9.4% vs. 2%), yet with similar 5-year OS (83% vs. 76%) and RFS (52% for both) [95]. Therefore, the right posterior sectionectomy is to be selected over the right hepatectomy in cases that allow complete tumor resection [89]. When the right posterior sectionectomy cannot ensure resectability, the systematic extended right posterior sectionectomy (SERPS) has been proved to be a feasible alternative to the right hepatectomy [24].

Liver failure risk and mortality are also increased due to an insufficient FLR in the setting of cirrhosis in patients with central tumors, for which extended right or left hepatectomy has been the standard recommended approach. An alternative is central or mesohepatectomy (S4–5–8 and middle HV resection), that preserves more parenchyma while ensuring complete tumor resection [89]. It has been shown that following a central hepatectomy, postoperative bilirubin levels >4 mg/dL are notably less common (2% vs. 39%) [96] and the liver failure risk is lower (1.7% vs. 10.6%) [97] than after an extended right/left hepatectomy; yet, the 5-year OS and RFS are similar [97]. As it is still a major hepatectomy, central hepatectomy is, however, to be avoided whenever possible. A feasible alternative is the systematic limited central hepatectomy [29].

The current practice involves performing parenchyma sparing, preferably anatomic LR, such as sub-segmentectomies, segmentectomies, bisegmentectomies, right posterior sectionectomy or central hepatectomy [89]. In case this is not feasible, non-anatomic LR such as SERPS [24] or systematic limited central hepatectomy [29] are options to be deployed whenever possible to avoid a major hepatectomy.

When comparing laparoscopic and open LR, DFS rates were not different in both groups, and overall survival rates were higher in the laparoscopic group (*p* = 0.033). The survival outcomes were comparable between laparoscopic and open LR in patients with stage 1 HCC; however, the laparoscopic approach provides better disease-free survival rate in patients with stage 2 HCC (*p* = 0.045). The difference is suggested to be caused by less blood loss and less tissue manipulation, expressed as “no-touch” in [98].

PVE and PVL are the most frequently used strategies before major LR in patients with HCC. Both are widely available, allowing a resectability rate of 75% [99]. The rationale of using sequential TACE and PVE is that the absence of arterial flow stops the tumor’s increase in size while waiting for hypertrophy after PVE [100]; another potential benefit is the higher hypertrophy rate compared with PVE on its own [101]. Sequential TACE and PVE survival is comparable to ALPPS and better than PVE or PVL [99].

ALPPS was associated with an increased mortality risk and a higher risk for developing liver failure [102,103,104,105] and showed similar OS results when compared to other strategies [99].

### 4.3. Intrahepatic Cholangiocarcinoma

LR is the only curative treatment option [106,107] for intrahepatic cholangiocarcinoma (ICC), despite the reportedly frequent locoregional recurrence, extrahepatic metastases and the low 5-year OS [108,109,110].

Anatomic and non-anatomic LRs are associated with comparable intraoperative bleeding and morbidity, but liver failure occurs more often following anatomic LR [111]. Non-anatomic LR has been linked with a higher rate of positive surgical margins, but this seemed to not impact the OS or the DFS [111]. However, it has also been shown that negative surgical margins are associated with a beneficial OS and PFS following resection for ICC [112]. Positive margins have been linked to inferior results in the long run and the OS and DFS proved to become gradually worse for a margin width >1 cm [113]. In this sense, R1 vascular resection is not recommended [114].

Non-anatomic LR has proved to be non-inferior to anatomic LR in terms of survival in the case of solitary ICC not invading contiguous organs or extrahepatic metastases, which shows that these patients, particularly in the context of cirrhosis, could benefit more from a non-anatomic hepatectomy, given the lower risk of liver failure [111]. 

ALPPS is also a valid indication for intrahepatic cholangiocarcinoma (IHCC) [115], with a 3-year OS of 21.4% and better results in the case of R0 resection and single lesions [115]. In the case of HCC, the 5-year overall survival was 46.8% [104,116].

### 4.4. Hilar Cholangiocarcinoma

Resectability of hilar cholangiocarcinoma is mainly dictated by the intrahepatic tumor extension. Bismuth–Corlette type III tumors are usually resectable by performing a hemi-hepatectomy that can be extended, while Bismuth–Corlette type IV tumors are surgically manageable only in selected patients. Other aspects that determine resectability are the tumor invasion of the portal vein and/or hepatic artery, FLR in terms of both volume and functional status as well as its competent biliary drainage and PVE feasibility. Regardless of resection extent, en bloc S1 resection is advised [117]. In patients presenting with jaundice, preoperative biliary drainage is recommended to maximize postoperative liver function and FLR regeneration. The R0 resection rate in Klatskin tumors can reach 92%, however, unsatisfactory postoperative morbidity rates of up to 54% have been reported [117].

Left liver resections are more beneficial as they allow sparing the right liver, hence left trisectionectomy is a feasible option for cases of Klatskin IV tumors that do not involve the right hepatic artery [117]. Mesohepatectomy should be taken into consideration when the bile ducts of S6–7 and S2–3 are not affected, and the portal and arterial branches of these sparable segments can be preserved [117].

Strategies to maximize liver functional status using PSS to reduce the extent of LR in Klatskin tumor have resulted in a lower overall mortality [117]. For example, in Bismuth–Corlette type IV tumors, when the joining level between the bile ducts of S4 and the biliary tree allows it, S4s could be spared during a PS extended right hepatectomy, replacing a right trisectionectomy [117]. For left lesions with restricted extension to sectorial ducts of S5–8 and S6–7, limiting the resection to part of S5–8 that results in a PS extended left hemi-hepatectomy should be considered [117]. Accurate evaluation of bilio-vascular anatomy and of potential invasion is necessary before considering parenchyma-sparing hepatectomies. In addition to preoperative imaging techniques, IOUS should be used as it allows real-time anatomy analysis as well as resection guidance.

A study [118] showed a mortality rate twice as high for patients that underwent ALPPS for Klatskin tumors than that of patients with a comparable hepatic volume that did not undergo ALPPS (48 vs. 24%). Median survival was lower in ALPPS than in the control group (6 vs. 29 months). Data show that Klatskin tumors may not be a common indication for ALPPS and its use should be limited.

### 4.5. Other Focal Liver Lesions

Indications for surgical resection of GIST include limited disease, progression refractory to TKI and locally advanced or previously unresectable tumors that manifest favorable response to neoadjuvant therapy with TKI [119].

In the case of NELM, LR is the treatment of choice whenever feasible, since patient outcomes after resection have been reported to be favorable compared to those with unresectable tumors [120]. Repeat hepatectomy, if feasible, can be a good option for intra-hepatic recurrence and can provide long-term survival [121].

Regarding hemangiomas, as complications are rare, observation is justified in the absence of symptoms. LR is indicated in patients with abdominal (mechanical) complaints or complications or when diagnosis remains inconclusive. Enucleation is the preferred surgical method according to existing literature [122].

ALPPS may be also deployed in neuroendocrine liver metastases (NELMs) [123], and other rare indications, such as lymphoma [124]. In NELM, 2-year overall survival rates of 95.2% were reported [123].

## 5. Conclusions

Parenchyma-sparing liver surgery, along with techniques of complex liver resection and intraoperative ultrasound guidance, is in our opinion currently the preferred strategy to treat liver tumors. Liver volume-manipulating regenerative liver surgery (portal vein embolization or ligation, two-stage hepatectomy, venous liver deprivation, ALPPS, etc.) should be applied when resectability needs to be extended beyond the possibilities of parenchyma-sparing liver surgery.

## Data Availability

Not applicable.
